# The effect of the Covid-19 pandemic on Tuberculosis (TB) case notification in Ogun State, Nigeria

**DOI:** 10.4314/ahs.v23i3.44

**Published:** 2023-09

**Authors:** Olusoji J Daniel, Janet O Bamidele, Adekunle D Alabi, Musibau A Tijani, Callistus A Akinleye, Kolawole S Oritogun, Festus O Soyinka, Olusola A Adejumo

**Affiliations:** 1 Department of Community Medicine and Primary Care, Olabisi Onabanjo University Teaching Hospital, Sagamu, Ogun State; 2 Department of Community Medicine and Primary Care, Faculty of Clinical Sciences, Olabisi Onabanjo University Sagamu Campus, Ogun State; 3 State Tuberculosis, Leprosy & Buruli Ulcer Control Programme, Department of Public Health, Ministry of Health, Abeokuta, Ogun State; 4 Department of Community Medicine, Osun State University, Osogbo; 5 Department of Community Health, Lagos State University Teaching Hospital Ikeja, Lagos

**Keywords:** Tuberculosis, COVID-19, case notification, private-public mix, private sector

## Abstract

**Introduction:**

COVID-19 pandemic has resulted in disruptions in delivery of Tuberculosis services especially, in resource-limited settings. Provisional data by the WHO from 84 countries indicates that about 1.4 million fewer people received care for tuberculosis in 2020 than in 2019. This study assessed the effect of COVID-19 pandemic on tuberculosis case notification rates in Ogun state, Nigeria

**Methods:**

A retrospective review of presumptive TB and diagnosed TB cases that were notified in 2019 and 2020. Analysis was done using Epi-info version 7.2.3.1. Level of statistical significance was p < 0.05

**Results:**

A total of 3102 and 3326 confirmed cases were reported in 2019 and 2020 respectively with an increase of 7.2%. There was significant decline in total number of cases notified in Q2, 2020 compared to 2019 (p=0.001) with a significant increase in proportion of TB cases notified by private facilities from 11.65% in 2019 to 20.27% in 2020.

**Conclusion:**

Total TB cases notified in Ogun state increased during the covid-19 pandemic. There was significant decline in TB cases during the lockdown but an increase in proportion of TB cases notified by private facilities demonstrating that private facilities can withstand disruptions to TB case notifications due to the Covid-19 pandemic.

## Introduction

COVID-19 is a disease of public health importance which first came into the limelight in December 2019 following an outbreak of a new coronavirus infection which caused acute respiratory syndrome of unknown origin.^1^

By March 11 2020, with the disease spreading to several other countries, WHO declared the disease a global pandemic. Since then, there has been a continuous rise in the number of daily cases of COVID-19 seen in countries worldwide with the developed countries being more severely hit by the disease than less developed countries. As of 23^rd^ May 2022, there were over 520 million confirmed cases of COVID-19 globally with more than 6 million deaths reported.^2^

The first case of COVID-19 was reported in Nigeria on the 27^th^ of February 2020^3^ and as of 23^rd^ May 2022, Nigeria now has over 255,000 confirmed cases of the disease with over 3000 deaths reported. The highest concentration of cases reported in Nigeria is in Lagos state with over 98,000 confirmed cases and 769 deaths. Ogun state has more than 5,500 confirmed cases and 82 deaths reported.^4^

Considering the high rate of transmission of the COVID-19 disease and the morbidity and mortality associated with the disease,^5^-^8^ countries needed to mobilize and deploy resources to combat the pandemic in their countries.^9^ Redeployment of staff and resources from other public health programs, and reduction in the number of patients attended to at outpatient and inpatient clinics were not uncommon in the affected countries.^9^ This resulted in the diversion of varying degrees of efforts from other endemic diseases of public importance such as tuberculosis (TB) in affected countries.^10^,^11^

Tuberculosis remains an issue of great public health importance to many developing countries including Nigeria. It is a disease that affects over 10 million people annually with 1.5 million deaths and has been in existence for millions of years.^12^ According to World Health Organization, Nigeria is one of the 30 countries with a high burden of Tuberculosis, multidrug-resistant tuberculosis and TB/HIV.^12^ In 2019, it was estimated that Nigeria, with a population of approximately 200 million had about 440,698 new cases of tuberculosis using the national incidence rate of 219/100,000^13^ out of which only 120,266 (27%) were notified and placed on treatment (2019 annual TB report). According to the Department of Planning Research and statistics in Ogun state, the estimated TB burden for Ogun state in 2019 and 2020 with a population of approximately 5,685,800 and 5873430 respectively was 12,451 and 12,863.

Over the years, control strategies have been implemented to reduce the incidence and prevalence of tuberculosis globally. The current global strategy aims to end tuberculosis by the year 2030. The targets of the strategy are to reduce tuberculosis deaths by 95%, reduce new cases by 90% between 2015 and 2035 and ensure that no family is burdened with catastrophic expenses due to tuberculosis.^14^ Before the onset of COVID-19, the progress towards achieving the 2020 milestone for this goal was slow with many high-burden countries lagging. This has now been worsened due to the emergence of the COVID-19 pandemic which has impinged negatively on already established control measures by disrupting routine health services and the resultant redistribution of scarce public health resources. A significant contributor to ending tuberculosis is adequate case notification and ensuring successful treatment outcomes. It has been predicted that the number of people dying from tuberculosis may increase by 0.2-0.4 million in 2020 alone in a case where the COVID-19 pandemic disrupts activities to the extent that case notification and treatment falls by 25-50% for 3 months.^14^

Therefore, this study aimed to determine the effect of COVID-19 on case notification and treatment outcomes in Ogun State with the ultimate goal of providing information for policymakers and stakeholders to make better decisions regarding case notification and treatment outcomes within the state.

## Methods

### Study Location

Ogun state is located in the South West region of Nigeria and shares a close boundary with Lagos and the Atlantic Ocean in the South, Oyo and Osun states in the North, Ondo state in the East and the Republic of Benin in the West. It has 20 local governments and three senatorial districts. The estimated population for the state for 2019 and 2020 is 5,685,800 and 5,873,430 respectively. The State has 426 primary healthcare facilities, 26 secondary healthcare facilities, three tertiary healthcare institutions and 904 registered private health facilities. The Ogun State Tuberculosis and Leprosy Control Programme became operational in 1993 and the Public-Private Mix for TB management commenced in Ogun state in 2007. There are, presently, about 120 facilities providing TB treatment services in all the 20 LGAs in the state. A total of 134 facilities (100 public and 34 private) reported TB patients at the end of 2014.

### Study design

The study was a retrospective review of TB cases notified to the Ogun State TB programme between 2019 and 2020. The data for the study was obtained from the routine programme data submitted quarterly to the Ogun state TB and Leprosy Control Programme.

### Data collection

The investigators retrieved case notification data from the quarterly case notification form of the Ogun state TB program into a proforma developed by the researchers. The data was then entered into the Epi info version 7.2.3.1 statistical software.

### Coordination of Tuberculosis control in Ogun State

TB control in Ogun State is coordinated by the Ogun State TB and Leprosy Control Programme (OGST-BLCP). The DOTS management of TB started in Ogun State in the year 2003. TB treatment and investigations (sputum for acid-fast bacilli and Gene Xpert) are free in the state; however, patients are required to pay for any other laboratory tests. Treatment duration is 6 months and the treatment regimen consisted of 2 months intensive phase using medications that include Rifampicin, Isoniazid, Pyrazinamide, and Ethambutol as a fixed-dose combination and 4 months continuation phase of rifampicin and isoniazid as a fixed-dose combination. Patients' treatment could either be supervised by health workers at the DOTS facility or by treatment supporters chosen by the patients. Patients are monitored during treatment using clinical and bacteriological methods. Clinically the weight of the patients is measured monthly while Sputum AFB is carried out at the end of two, five and six months of treatment. To reduce loss to follow-up, patients diagnosed with TB are managed at the DOTS facility nearest to their residence.

All presumptive TB clients are offered human immunodeficiency virus (HIV) tests following the HIV testing and counselling policy in the country. Determine (Alere Determine HIV-1/2 test, Matsuhidai Matsudo-shi, Chiba-ken, Japan) and Uni-Gold (Trinity Biotech PLC, Wicklow, Ireland) are used in the parallel algorithm. STAT-PAK (Chembio diagnostic systems Inc., Medford, Newyork, USA) is used as a tie-breaker if a discordant result is obtained. The HIV test is done free of cost for patients, and TB/HIV co-infected patients are offered co-trimoxazole in addition to anti-TB drugs.

Each DOTS facility is managed by a TB focal person who is responsible for the day-to-day running of the facility and for ensuring that patient records are intact. The records of all TB patients in DOTS facilities within an LGA are sent to the OGTBLCP by the LGA TB supervisor at the end of every quarter.

### Definitions

According to the standard definitions of the National Tuberculosis and Leprosy Control Program guideline (NLCP) adopted from WHO, the following clinical case and treatment outcome definitions were used.

### Pulmonary TB, Smear-positive

A patient with at least two sputum specimens which were positive for acid-fast bacilli (AFB) by microscopy.

Pulmonary TB, Smear-negative

A patient with symptoms suggestive of TB, with at least two sputum specimens which were negative for AFB by microscopy, and with chest radiographic abnormalities consistent with active pulmonary TB (including interstitial or miliary abnormal images).

### Extrapulmonary TB (EPTB)

This includes tuberculosis of organs other than the lungs, such as lymph nodes, abdomen, genitourinary tract, skin, joints, bones, and meninges. Diagnosis of EPTB based on fine needle aspiration cytology or biochemical analyses of cerebrospinal/pleural/ascitic fluid or histopathological examination or strong clinical evidence consistent with active extrapulmonary tuberculosis, followed by a decision of a clinician to treat with a full course of antituberculosis chemotherapy.

### Ethical issues

The data to be used for this study was retrieved from secondary aggregated de-identified data routinely collected by the Ogun state Tuberculosis and Leprosy Control Programme. Therefore, ethical approval was not required. However, verbal and written permission was obtained from the Ogun state Ministry of Health to utilize the required data.

### Data analysis

The data were analysed using the Epi info software version 7.2.3.1. Percentages of numerical variables were determined and the Chi-Square test was used to compare proportions of independent variables. P values less than 0.05 were considered statistically significant.

## Results

**Figure 1:**
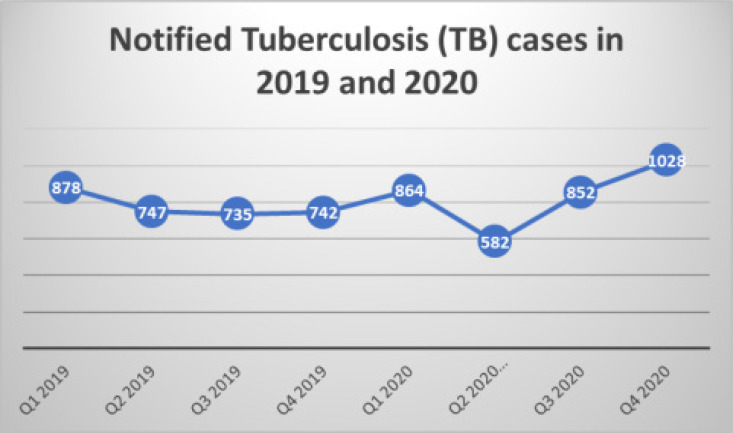
Quarterly TB Notification in Ogun State in 2019 and 2020

**Figure 2:**
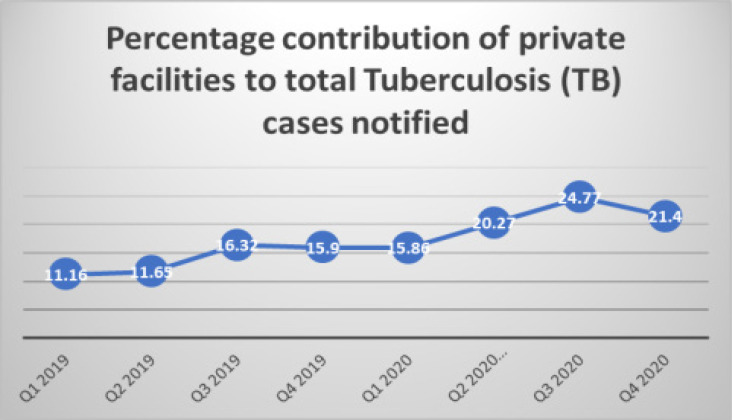
Percentage contribution of private facilities to TB case notification in Ogun State in 2019 and 2020

There was a 41.4% increase in the number of presumptive TB cases notified in 2019 (17,334) compared to the year 2020 (24,516) as seen in Table 1. However, there was only a 7.2% increase in the number of TB cases notified from 3102 in 2019 to 3326 in the year 2020 as shown in Table 2. The quarterly TB cases notified in quarter 1, 2019 was 878. This declined slightly to 747 in quarter 2, 2019 and was relatively stable until quarter 1, 2020 when 864 cases were notified. This declined by about 32.6% to 582 in quarter 2, 2020 and thereafter increased to 1028 in quarter 4, 2020 as described in Figure 1. Table 3 shows the facilities in Ogun State, Nigeria. There was a statistically significant quarterly increase in the number of TB cases notified by private facilities in 2020 compared to 2019. The percentage increase in TB cases increased from 11.16% in quarter 1, 2019 to 21.4% in quarter 4, 2020 as shown in Figure 2.

**Table 1:** Total number of Presumptive TB cases reported in Ogun State Nigeria for the years 2019 and 2020

Quarter	Year 2019 n (%)	Year 2020 n (%)	P values
Q1	4863	5532	0.001
Q2	4993	2881	0.001
Q3	3686	6342	0.001
Q4	3792	9761	0.001
**Total**	17334	24516	

**Table 2:** Total number of confirmed TB cases reported in Ogun State Nigeria for the years 2019 and 2020

Quarter	Year 2019 n (%)	Year 2020 n (%)	P values
Q1	878	864	0.038
Q2	747	582	0.001
Q3	735	852	0.070
Q4	742	1028	0.004
**Total**	3102	3326	

**Table 3:** Proportion of total TB cases reported by private facilities in Ogun state Nigeria for the years 2019 and 2020

Quarter	Total number of TB cases reported in 2019	Total number of TB cases reported in 2020	Proportion reported by private facilities in 2019 (%)	Proportion reported by private facilities in 2020 (%)	P values
Q1	878	864	98/878 (11.16)	137/864 (15.86)	0.005
Q2	747	582	87/747 (11.65)	118/582 (20.27)	0.001
Q3	735	852	120/735 (16.32)	211/852 (24.77)	0.001
Q4	742	1028	118/742 (15.9)	220/1028 (21.40)	0.004
**Total**	3102	3326			

## Discussion

Globally, the covid 19 pandemic has had an impact on achievements made in the control of tuberculosis over the years, especially in terms of case notification. Globally, there was a large fall from 7.1 million cases notified in 2019 to 5.8 million cases in 2020 and this fell below the expected 10 million people who developed TB in 2020.^15^ The study shows that there was an increase in the number of presumptive TB cases by about 41% between 2019 and 2020. However, the number of diagnosed TB cases notified increased by about 7.2% during the same period. This may be because both COVID-19 and TB presented with similar symptoms such as cough, fever and difficulty in breathing and only a fraction of those who presented with symptoms was diagnosed with TB which suggests that a large majority of the presumptive TB cases may have other respiratory infections including covid-19.^16^ Also, some of the diagnosed TB patients may have co-infection with COVID-19, especially in the early phase of the pandemic where access to diagnosis of COVID-19 infection was limited. COVID-19/TB co-infected patients are more likely to have severe diseases and die.^17^ It is therefore imperative to screen TB patients for Covid-19.

Despite the global pandemic in 2020, our study showed an increase in the number of TB cases. This is in contrast to what was reported in the United States where there was a 20% decrease in cases of TB notified in 2020 compared to the previous year.^18^ A study conducted in Vietnam also reported that overall TB case notification dropped by 8% in 2020 compared with 2019.^19^ Likewise, in Malawi a 24% overall decrease in case notification was reported in 2020.^20^ One of the plausible reasons for the increase in TB cases in the state during the pandemic may be as a result of the significant increase in the proportion of TB cases notified by the private facilities in the year 2020. It is important to note that despite the lockdown period which began in Q2 of 2020, the proportion of TB cases reported by the private facilities in Q2 of 2020 was still higher than that reported in 2019. The contributions of private facilities to TB case notification in the high TB burden countries have ranged from 5% in 2012 to 28% in 2019.^21^

This has helped to close some of the missing gaps in the TB case notification and emphasized the importance of multi-sectoral collaboration in Ending TB.^22^ The private sector in many countries has stepped up to fill in the gap during this pandemic by implementing several innovative strategies such as active case finding, home delivery of medications, transport support, telephone follow up and telemonitoring of treatment adherence.^23^ This engagement of the private sector should be sustained to develop a more resilient health system in the country as it has been shown in this study that the private sector can withstand sudden disruptions such as the pandemic within the health care system.

There was a decline in the number of cases notified during Q2 of 2020 compared to the same period in the year 2019. The period coincided with the emergence of the first case of COVID-19 in the country in the late first quarter which led to a nationwide lockdown of all activities in the country. This aligns with what was found in Vietnam where there was a 29% decrease in case notification in the second quarter of 2020 compared with the same period in 2019.^19^ This lockdown resulted in a decline in the number of people seeking services, especially in the public health sector. Factors such as difficulty accessing healthcare services 24,25 and fear of catching COVID-19 at the healthcare facilities26 have been identified as contributors to the reduced case notification observed during this period by some studies.

The lockdown rules were relaxed in the third and fourth quarters of 2020, and this possibly accounted for the increase in the number of presumptive TB cases notified. The finding from this study is similar to what was found in China and Vietnam where there was a rebound in TB notification in the months after the COVID-19 restrictions were eased.^19^,^27^ The increase in presumptive and diagnosed TB cases observed in this period may be due to an increase in the number of people infected with TB as a result of the continued transmission of TB in the community due to the lockdown. Many individuals were at home during the lockdown which could have increased indoor transmission of the bacteria because presumptive TB patients could not access services. As a result of this, patients may present late and possibly with advanced stages of the disease with a consequent unfavourable treatment outcome and prognosis. More studies are needed to explore the effect of COVID-19 on the treatment outcome of tuberculosis patients in the state.

## Limitations

This study is a retrospective study and there may be some seasonal fluctuations which affected the number of cases notified within the study period. In addition, the study assumed that all other factors such as environmental, health systems, behavioural and social factors remained the same except for the COVID-19 pandemic in the period that was studied.

## Conclusion

The present study shows that in Ogun state, the total number of TB cases reported in the year 2020 increased compared to 2019. It also shows that there was a significant decline in TB case notification during the second quarter of 2020 with an increase in the proportion of cases notified by the private facilities. It highlights the importance of the Public-Private Mix (PPM) to improve TB case notification, especially in low-income countries with weak health systems, inadequate resources and a high TB burden. There is a need to continue to strengthen the collaborative network between the private and public sectors involved in TB control in the country to develop a resilient healthcare system that can withstand unanimated shock in the health care system.
